# The enduring echoes of juvenile bullying: the role of self-esteem and loneliness in the relationship between bullying and social media addiction across generations X, Y, Z

**DOI:** 10.3389/fpsyg.2024.1446000

**Published:** 2024-08-02

**Authors:** Sabina Lissitsa, Maya Kagan

**Affiliations:** ^1^School of Communication, Ariel University, Ariel, Israel; ^2^School of Social Work, Ariel University, Ariel, Israel

**Keywords:** social media addiction, juvenile bullying, self-esteem, loneliness, masculinity, Generation X, Generation Y, Generation Z

## Abstract

**Objective:**

Being bullied is a profoundly distressing experience for children and adolescents, with the potential for adverse mental and behavioral outcomes throughout their adult years. This study aims to explore the association between juvenile bullying, self-esteem, loneliness, and social media addiction among men across three generational cohorts: X, Y, and Z.

**Method:**

The study utilized an online survey, administering structured questionnaires to 797 men aged 18–58 divided into three generational cohorts: 142 individuals from Gen X (born between 1965 and 1980), 275 from Gen Y (born between 1981 and 1996), and 380 from Gen Z (born between 1997 and 2005).

**Results:**

The findings demonstrate that across all three generations, there was a positive correlation between experiencing bullying in one’s youth and social media addiction in adulthood. Among Gen X, self-esteem did not act as a mediator in this relationship, nor did loneliness moderate the links between bullying and social media addiction, or between self-esteem and social media addiction. However, for Gen Y and Z, self-esteem was found to mediate the relationship between bullying and social media addiction. Loneliness moderated the association between self-esteem and social media addiction in Gen Y and the association between bullying and social media addiction in Gen Z.

**Conclusion:**

The differences observed among generational cohorts can be attributed to changes in masculinity norms, the evolution of bullying types, and the rapid development of social media platforms, catering to the distinct needs and gratifications of each generation.

## 1 Introduction

Being bullied ranks among the most upsetting and damaging experiences for children and adolescents. Subjection to acts of bullying, whether verbal or physical, mental or social, has a strong long-term impact on victims, such as the risk of low self-esteem, depression (Jochman et al., 2017; Wang et al., 2023) and impaired social functioning (Sandoval et al., 2015; Rehman et al., 2022). These negative outcomes may frequently lead to problematic digital habits, with victims using social media as a coping mechanism or escape, potentially leading to addiction. This reliance on social media may lead to reduced real-world interactions (Apriyani, 2023) and compulsive behavior (Van den Eijnden et al., 2016), worsening the initial effects of bullying.

Social media addiction is marked by a growing urge to use these platforms, neglecting life duties and feeling anxious or irritable when usage decreases or ceases (Van den Eijnden et al., 2016; Casale et al., 2018; Sun and Zhang, 2021; Fournier et al., 2023). This addiction may be particularly pronounced in individuals with a history of being bullied, suggesting a complex interplay between past trauma and present offline and online behavior (Vessey et al., 2022). Numerous studies have focused on the growing issue of addictive social media use, investigating its causes and effects (Aksoy, 2018; Hou et al., 2019; Al-Samarraie et al., 2021; Treviño Benavides et al., 2023; Hernández et al., 2024). However, research specifically examining the link between juvenile bullying experiences and the prevalence of social media addiction in adulthood has been relatively scarce. Furthermore, the fact that this issue has not been explored among adult men underscores a significant gap in our understanding of the diverse effects of bullying across genders. Given the increasing role of social media in contemporary society, clarifying the distinct challenges experienced by men in this context is crucial for a more comprehensive understanding of the enduring consequences of childhood bullying. By examining how men respond to such adversities, a deeper comprehension of the long-term effects of childhood bullying can be attained. Such knowledge may contribute to efforts to develop interventions that target the diverse needs of the male population and ultimately foster a supportive environment for men who are suffering from the consequences of bullying experienced during their formative years.

Self-esteem is commonly regarded as a crucial factor in the dynamics of bullying, although most studies treat bullying victimization as an outcome variable, suggesting that individuals with low self-esteem attract negative attention from peers, provoking specific bullying behaviors from others (Olweus, 1992) or are less capable of defending themselves from bullies’ attacks (Hodges and Perry, 1999). Bidirectional relationships between these variables have also been reported (Boulton et al., 2010). However, comparatively few studies have investigated the effect of childhood bullying victimization on self-esteem in adulthood (Schäfer et al., 2004). Moreover, the complex dynamic of bullying victimization in childhood and self-esteem and social media addiction in adulthood is totally overlooked by the literature. Understanding this dynamic is crucial, as it sheds light on the long-term psychological impact of bullying and its potential role in shaping behaviors, including compulsive behaviors, and vulnerabilities, later in life.

Loneliness, which is characterized by an intensified sense of isolation and an unfulfilled need for meaningful social connections, also plays a pivotal role in shaping individuals’ interactions with social media platforms (Schäfer et al., 2004; Matthews et al., 2022). Higher levels of loneliness may potentially act as a catalyst that magnifies the impact of past negative social experiences and of low self-esteem on addictive digital behaviors. Thus, lonely individuals might turn to social media as a way to satisfy their need for connection and compensate for previous emotional shortcomings (Rachubińska et al., 2021).

### 1.1 The current study

Because of the evolution of masculinity from a hegemonic stance (Connell et al., 1985) to a hybrid approach (Bridges and Pascoe, 2014), broadening the scope of male behaviors and emotional expressions, it is necessary to address bullying within the context of generational differences, as this shift impacts how bullying is experienced and managed by men from different generational cohorts. Bullying has also been influenced by cultural and technological shifts, transitioning from traditional physical and verbal forms to cyberbullying (Johansson and Englund, 2021), with concomitant changes in gender norms, attitudes, and societal expectations. Accordingly, the *purpose of the current study* was to examine the interrelationships between the experience of bullying in childhood and adolescence, and self-esteem, loneliness and social media addiction among adult men from three generational cohorts: Gen X, born between 1965 and 1980; Gen Y, born between 1981 and 1996 and Gen Z, born after 1997 (Roth-Cohen et al., 2022). Examining these interrelationships across different generations of men can provide valuable insights into the changing dynamics of bullying, its long-term psychological effects, and the role of rapidly evolving technological landscapes.

## 2 Literature review

### 2.1 Bullying, self-esteem and media addiction

Bullying is characterized as a deliberate and repetitive act of aggression, whether physical, verbal, or psychological, executed by an individual or a group of individuals with more power or numerical superiority against a peer perceived as vulnerable or different (Kaltiala-Heino et al., 2000). Bullying behavior falls into four general categories: direct–physical (for example, assaults or theft), direct–verbal (threats, insults, or name-calling), indirect–relational (social exclusion and spreading nasty rumors), and the newest form, cyber (Kagan et al., 2018; Wu et al., 2022). Cyberbullying is defined as the repeated use of online channels, such as mobile phones or other electronic devices, to threaten, mock, or abuse others, causing them pain (Hinduja and Patchin, 2014). Common forms of cyberbullying include stalking, virtual harassment, leaking personal data, hate speech, creating false identities, and ostracizing others (Slonje et al., 2013). The dangers are heightened by the ease of sharing text, images, voice messages, videos, and animations, which spread uncontrollably and cause emotional harm (Li et al., 2021). The lack of privacy and boundaries in cyberbullying increases the risk of psychological, emotional, and social violence, making individuals more vulnerable to becoming “victims of the virtual world” (Gohal et al., 2023). Bullying often targets individuals perceived as not conforming to dominant social norms (Davis et al., 2015). Boys who fall prey to bullying often exhibit signs of physical vulnerability, emotional sensitivity, and social exclusion, thereby heightening their susceptibility to victimization (Hong et al., 2018).

*Social Learning Theory* suggests that individuals learn social behaviors by observing and imitating others, as well as from the outcomes of these behaviors (Bandura, 1985). Experiences of bullying during their formative years can teach individuals negative behavioral patterns and coping mechanisms (Waasdorp and Bradshaw, 2011). In response to the negative experiences of bullying, they might learn to seek social validation and approval in order to maintain their social status or perhaps to avoid the stigma associated with being a victim (Oransky and Marecek, 2009). As adults, individuals who were bullied as juveniles might model their social behaviors on these learned patterns. They might turn to social media as a safe space (Mandel et al., 2017), where they can control their interactions and avoid the direct negative social experiences they encountered in childhood and adolescence. Social media platforms provide an environment where they can observe and imitate behaviors that appear to receive social validation and approval. This can include curating a desirable online persona or engaging in specific online interactions that seem to yield positive feedback and attention. However, for adults seeking to compensate for earlier negative peer experiences, this reinforcement can be addictive. Wegmann et al. (2022) found that users seek gratification and compensation for negative states through addiction-related behaviors (internet addiction, compulsive buying, gaming addiction, social media addiction).

Social media addiction refers to a maladaptive psychological dependency on social media and manifests in a strong emotional attachment to social media, constant worries about online activities, as well as a strong uncontrollable need to be permanently online, despite potential problems in other areas of daily life (Casale et al., 2018; Tarafdar et al., 2020; Jabeen et al., 2023). A key distinction between normal over-engagement in social media and social media addiction is that the latter is associated with unfavorable consequences, such as negative implications for individuals’ mental health and well-being (Jelenchick et al., 2013; Ozimek et al., 2024). Although social media addiction has not been officially classified as a behavioral disorder as are gaming and gambling (Brand et al., 2019), it shares similar symptoms, causing individuals to neglect work and home responsibilities in favor of social media (Zivnuska et al., 2019; Cao et al., 2020; Yayman and Bilgin, 2020).

The instant gratification and perceived control over their social environment on these platforms can lead to excessive use, as it fulfills a learned need for social validation (Wadsley et al., 2022). This tendency to seek social rewards and alleviate negative affect states through social media use (Wegmann et al., 2022) suggests that experiences of bullying in youth, which often result in a heightened need for social approval and relief from negative states, could plausibly be linked to social media addiction in adulthood. Accordingly, we formulate H1:

*H1*: Subjection to bullying as juveniles will be positively associated with social media addiction in adulthood among men across all generational cohorts.

In terms of Rogers (1959) *Self-Concept theory*, experiences of bullying as juveniles can be seen as form of conditional positive regard, where acceptance and affection from peers are contingent upon certain conditions. This can lead to the development of a self-concept based on these conditions, often internalizing the negative perceptions from bullying experiences. As a result, individuals may develop what Rogers referred to as conditions of worth, which can significantly lower their self-esteem. As adults, individuals with a history of being bullied might experience incongruence between their self-concept (shaped by past bullying) and their actual experiences. This incongruence can manifest as low self-esteem, where there’s a disparity between their perceived self (often negative due to bullying) and their ideal self (what they wish to be). There is ample evidence that the detrimental effects of childhood bullying, including increased social anxiety, isolation, low self-esteem and shame issues, difficulties in relationships and trust, continue long after victimization has ended (Boden et al., 2016; deLara, 2022). The drive to resolve this incongruence and to achieve a sense of positive self-worth (Nelson-Jones, 2000) can lead adults to turn to social media. Social media platforms may provide these individuals with conditional positive regard in the form of likes, comments, and followers, which temporarily reduces the incongruence in their self-concept. This may produce what Rogers (1961) termed a “fully functioning person” but in a limited, digital context, wherein the person feels more valued, understood, and accepted online than in offline reality. The platforms offer an environment where adults can continuously seek and often receive the positive regard they feel they lack. This reliance on social media for conditional positive regard can become addictive (Kardefelt-Winther, 2014). Accordingly, we formulate H2:

*H2*: Among all generational cohorts, self-esteem will mediate the positive association between subjection to bullying as juveniles and social media addiction, such that more intensive subjection to bullying will be negatively correlated with men’s self-esteem, which in turn will be negatively correlated with social media addiction.

### 2.2 Loneliness as a moderator in the relationships between bullying, self-esteem and social media addiction

Loneliness is defined as an emotional response to the perceived discrepancy between one’s actual and desired social relationships (Heinrich and Gullone, 2006). It emerges when individuals perceive a deficit in their social connections, whether it be in terms of quality or quantity (Hyland et al., 2019), or feel misunderstood and believe that their relationships lack meaning (Lim et al., 2016). Loneliness has been identified as a concept influenced by social norms and gender values (Franklin et al., 2019). Men are less willing than women to disclose and report feelings of loneliness, because they perceive such admissions as detrimental to the masculine image (Ong et al., 2016).

In a society that highly values social connectivity and networking (Sonnenberg and Sonnenberg, 2014), loneliness accentuates a state of isolation, starkly contrasting with the expected norms of continual social engagement and interaction. For lonely individuals, the need for social validation is intensified (Van Tilburg, 2021). Lacking substantial real-world social interactions, they may increasingly depend on social media to satisfy their need for social connection and approval. This dependency is heightened by their low self-esteem, driving them to continually seek affirmation in an environment where feedback is immediate and quantifiable (Ilies et al., 2007). Such dependency can escalate into addiction, particularly when real-life social needs are unmet (Hawi and Samaha, 2017). The accessibility and ease of obtaining positive reinforcement online can lead to a preference for digital interaction over real-world engagement (Laghi et al., 2013), further entrenching addictive behavior.

Adults who were bullied as juveniles and experience high levels of loneliness in adulthood may be more vulnerable to social media addiction (Vazsonyi et al., 2016; Rachubińska et al., 2021). The combination of unresolved trauma from the past and current feelings of isolation can lead individuals to seek comfort, validation, and a sense of belonging through social media platforms (Andalibi et al., 2016; Cauberghe et al., 2021). The varying experiences of bullying and loneliness as they relate to social media addiction highlight the need to consider how these issues manifest across different generations, each shaped by distinct societal and technological landscapes during their formative years.

### 2.3 Generational cohort theory

Introduced by Inglehart (1977), Generational Cohort Theory segments populations based on generational groupings, typically spanning 20–25 years or more, defined by the time it takes for a birth group to grow, mature, and reproduce within a society. This theory posits that generational cohorts, during their formative years, share key economic, social, and political experiences (Strauss and Howe, 1991), shaping their values, preferences, attitudes, and behaviors. These collective experiences forge a generational identity that tends to persist throughout the cohort’s lifetime (Inglehart, 1977; Pekerti and Arli, 2017).

#### 2.3.1 Generation X (born between 1965 and 1980)

The early 1980s and 1990s, the formative years of Gen X members, were a period of considerable economic and social uncertainty, as reflected in economic recessions, higher divorce rates and the spread of AIDS (Lissitsa, 2024). Whether households were split by divorce or both parents worked full time, children had to mature and become independent more rapidly. From an early age, boys were exposed to hegemonic masculinity messages (Connell et al., 1985; Lissitsa and Laor, 2021). The societal pressure on boys and men to adhere to aggressive norms, symbolized by sayings like “Do not cry” and “Bros before hoes,” relentlessly drove home the “Be a man” ethos. For Gen X men, the profound impact of childhood or adolescent bullying on self-esteem (deLara, 2022) was exacerbated by stringent masculinity norms, where admitting to such experiences or their emotional effects was often stigmatized as a sign of weakness (Lavorgna, 2021).

Gen X witnessed the advent of the internet and social media as adults (Çoklar and Tatli, 2021). This later exposure means their identity and self-esteem were less influenced by digital platforms during their formative years. Therefore, their interaction with social media as a coping mechanism might be limited due to their later life exposure to these platforms. Their sense of masculinity, influenced by hegemonic masculine norms (Connell et al., 1985; Kagan, 2024), might discourage them from acknowledging or acting upon feelings of loneliness, especially in the context of social media usage. Accordingly, we formulate H3:

*H3*: Among Gen X, loneliness will not serve as a moderator in the association between social media addiction and both antecedent variables: subjection to bullying in juvenile ages (H3a) and self-esteem (H3b).

#### 2.3.2 Generation Y – millennials (born between 1981 and 1996)

In contrast to Gen X, the formative years for Gen Y members coincided with economic growth. Gen Y witnessed a significant shift toward acknowledgment of the importance of emotional intelligence, a redefining of strength, allowing vulnerability, and a rejection of rigid gender norms (Roberts, 2018). Gen Y men were increasingly encouraged to move past traditional stereotypes assigned to them, leading to the formation of new identities not previously accepted or recognized in social and societal contexts. Millennials redefined traditional male gender roles, recognizing that men possess the same emotional spectrum as women and can gain from expressing their emotions (Green and McClelland, 2019). In “I Find That Offensive!” (2016), British writer Claire Fox defines Gen Y as the “Snowflake Generation.” She characterizes Gen Y individuals as overly sensitive, fragile, and ill-equipped to handle frustration or life’s challenges, citing excessive protection as the cause of their impaired ability to develop fully and manage real-life situations effectively.

Generation Y was the first cohort to grow up with the Internet and social media. They were the early adopters of Facebook and Instagram which they made into integral parts of their social lives (Giarla, 2019). These platforms are heavily oriented toward social connection, self-presentation, and the sharing of personal life and achievements, providing users with immediate feedback (likes, comments). For Gen Y men experiencing higher levels of loneliness, these platforms may become crucial for social interaction and validation. Given the heavy reliance of Gen Y on these platforms for social interaction, and the nature of Facebook and Instagram to foster a sense of connection, individuals with higher loneliness levels are more likely to form a dependency on these platforms to fulfill their social needs, enhancing the association between self-esteem and social media addiction. Moreover, in these platforms individuals can control their image and interactions, which is appealing for those with a history of bullying. Higher loneliness can intensify the need to seek validation and positive social interactions online, making these platforms particularly appealing and potentially addictive for individuals who are lonelier and seeking to compensate for social deficits or past traumas. Accordingly, we formulate H4:

*H4*: Among Gen Y, loneliness will moderate the association between self-esteem and social media addiction, such that this association will be more distinct at higher levels of loneliness (H4a). Loneliness will moderate the association between subjection to bullying as juveniles and social media addiction in adulthood, such that this association will be more distinct at higher levels of loneliness (H4b).

#### 2.3.3 Generation Z (born in 1997 and after)

Gen Z’s formative years were shaped by numerous recessions and financial upheavals, conflicts and terrorism risks, political instability, and the omnipresent influence of social media. This context understandably leads Gen Z to tend to exhibit pessimism and anxiety about their future, coupled with diminished trust in others (Adamy, 2018).

In an era that embraces a more inclusive view of masculinity, Gen Z has matured with the freedom to express their individuality in ways that reflect their unique experiences (Foster and Baker, 2022; Kosar et al., 2023). Born into an era where digital technology was already prevalent, Gen Z is inherently a digital generation (Kagan and Lissitsa, 2023). However, the frequent use and familiarity with social media also exposes them from childhood to risks associated with rising online violence, such as cyberbullying (Tirocchi et al., 2022). The digital world poses significant risks for the younger generation, despite its many opportunities (Umar et al., 2024). Gen Z believe they can be exposed to cyberbullying on social media at any time and that their likelihood of becoming cyber victims increases with the intensity of their social media usage (Erdoğdu and Koçyiğit, 2021).

They extensively utilize social media, streaming services, and platforms like YouTube and TikTok for content consumption and creation (Laor and Galily, 2022; Hermawan et al., 2023; Stahl and Literat, 2023). Social media platforms, offering both short-form and long-form content, serve as vital outlets for Gen Z men, supporting their engagement with hybrid masculinity, which embraces creativity and expressiveness. These platforms allow for the creation of personalized content and narrative control, providing Gen Z men, particularly those with past bullying experiences, with opportunities for self-expression and self-esteem rebuilding (Morris and Anderson, 2015). Gen Z individuals may use social media to post updates, showcase achievements, connect with like-minded peers, and receive instant feedback and validation, all of which may contribute positively to their self-esteem (Steinsbekk et al., 2021). The aim of these platforms to foster engagement and provide a sense of community means that even those who are lonely can find spaces where they feel valued and understood (Gatti and Procentese, 2021), thereby bolstering their self-esteem. This dynamic suggests that, for Gen Z men, the use of social media for self-esteem enhancement may occur independently of their loneliness levels.

Individuals subjected to bullying during their formative years often carry emotional scars, such as anxiety and trust issues, into adulthood (Schäfer et al., 2004). When these individuals also experience high levels of loneliness, their need for social connection and validation becomes more acute. The anonymity of online interactions, which reduces the social risks of rejection and further victimization, can be particularly appealing (Wójcik and Rzeńca, 2021). Social media platforms, with algorithms that promote continuous engagement by aligning content with user interests, can substitute for real-life interactions, especially for those experiencing loneliness (Rach and Peter, 2021). Gen Z’s unique digital nativity and their online relationship-building make them especially susceptible to these dynamics. Therefore, the combination of past bullying experiences and current loneliness intensifies the drive to seek solace and validation through social media, making the association between bullying and social media addiction more pronounced at higher levels of loneliness.

Accordingly, we formulate H5:

*H5*: Among Gen Z, loneliness will not moderate the association between self-esteem and social media addiction (H5a). Loneliness will moderate the association between subjection to bullying as juveniles and social media addiction in adulthood, such that this association will be more distinct at higher levels of loneliness (H5b).

## 3 Methods

### 3.1 Research population and sample

The study comprised 797 male participants aged 18 and above (range 18–58), divided into three generational cohorts: 142 individuals from Gen X (born between 1965 and 1980), 275 from Gen Y (born between 1981 and 1996), and 380 from Gen Z (born between 1997 and 2005) (Lissitsa, 2024). Table 1 displays the demographic characteristics of the study samples.

**Table 1:** Demographic characteristics of the samples (Gen X, Y, Z).

Variables	Gen X (*n* = 142)			Gen Y (*n* = 275)			Gen Z (*n* = 380)		
	Mean	SD	*N* (%)	Mean	SD	*N* (%)	Mean	SD	*N* (%)
Age	51.53	5.49		31.87	4.64		23.01	2.28	
Schooling level (years)	16.65	3.32		14.38	2.51		12.45	1.15	
Relationship status									
In a relationship			130 (91.5)			208 (75.9)			130 (34.3)
Not in a relationship			12 (8.5)			67 (24.1)			250 (66.7)
Religiosity									
Religious			90 (63.4)			171 (62.2)			254 (66.8)
Not-religious			52 (36.6)			104 (37.8)			126 (33.2)
Socio-economic status									
Low			3 (2.1)			16 (5.9)			22 (5.8)
Moderate			67 (47.2)			181 (66.3)			198 (52.1)
High			72 (50.7)			76 (27.8)			160 (42.1)

### 3.2 Sampling methods and procedure

The research protocol was reviewed and approved by the Institutional Committee for Nonclinical Research on Human Subjects at Ariel University, ensuring compliance with ethical guidelines. The link to a structured questionnaire was widely distributed across various online platforms, such as Facebook, WhatsApp groups and email. This method of distribution formed a non-probabilistic sample, however the extensive outreach contributed to a relatively large sample size, thereby broadening the spectrum of respondents represented within the study. Prior to engaging in the survey, all potential participants were asked to sign an informed consent form outlining the study’s objectives, procedures, and the confidentiality of their responses. Given the online distribution method employed, it was unfeasible to ascertain the precise number of individuals who accessed the survey link but chose not to participate. Consequently, it was not possible to establish a comprehensive response rate.

### 3.3 Measures

*Subjection to bullying in childhood and adolescence* - was assessed utilizing the Child Adolescent Bullying Scale (CABS; Strout et al., 2018). For the purposes of the current study, two adjustments were made to this scale. First, the items were formulated in the past tense, as they pertain to the childhood experiences of adult men. Second, the introduction to the questionnaire was adapted to encompass various types of bullying and worded as follows: “The following items relate to how your peers (referred to hereafter as kids/students) treated you during specific periods of your childhood/ adolescence, either directly or through digital communication tools like social media, websites, text messages, or other online platforms.” Subsequently, the participants were prompted to express their level of agreement with 20 specific statements about their encounters with bullying during their earlier years. For example: “Kids tried to make me feel bad on purpose”; “Kids posted or texted mean or hurtful messages, comments, or photos about me online”; “Kids at my school talked behind my back, shared my secrets, or spread rumors about me.” Participants were requested to respond on a Likert scale ranging from 1 (do not agree at all) to 5 (strongly agree). To derive the overall index score, the responses to all 20 items were totaled, resulting in scores that ranged from 20 to 100, with higher cumulative scores signifying increased subjection to bullying during the individual’s childhood or adolescence. Cronbach’s alpha coefficients were as follows: 0.962 for Gen X, 0.969 for Gen Y, and 0.973 for Gen Z.

*Self-esteem*- was assessed utilizing the ten-item Rosenberg Self Esteem Scale Revised (RSES; Rosenberg, 1965). Participants were requested to respond on a scale ranging from 0 (strongly agree) to 3 (strongly disagree) to each item. To derive the overall index score, the responses to all ten items were totaled, resulting in scores that ranged from 0 to 30, with higher cumulative scores signifying higher levels of self-esteem. Cronbach’s alpha coefficients were as follows: 0.897 for Gen X, 0.861 for Gen Y, and 0.836 for Gen Z.

*Self-reported loneliness*- was assessed utilizing a three-item scale (Hughes et al., 2004) that inquired about the frequency with which respondents experienced feelings of lack of companionship, being excluded, and being socially isolated. This scale used a range of responses from 1 (hardly ever), 2 (some of the time), to 3 (often) to capture the frequency of these experiences. To derive the overall index score, participants’ responses to these three items were summed, resulting in composite scores that could range from 3 to 9 with higher scores signifying a heightened level of loneliness experienced by the respondents. Cronbach’s alpha coefficients were as follows: 0.822 for Gen X, 0.795 for Gen Y, and 0.707 for Gen Z.

*Social media addiction* was assessed using the Bergen Social Media Addiction Scale (BSMAS). This scale comprised six Likert-type items, with respondents rating their experiences on a scale ranging from 1 (very rarely) to 5 (very often). The scale was designed based on the foundational characteristics of addiction, encompassing salience, mood modification, tolerance, withdrawal, conflict, and relapse as outlined by Griffiths (2005). The total BSMAS index was derived by summing up the responses to all six items, resulting in scores that ranged from 6 to 30. Higher scores on this index indicated increased levels of social media addiction among the respondents. Cronbach’s alpha coefficients were as follows: 0.909 for Gen X, 0.900 for Gen Y, and 0.868 for Gen Z.

Additionally, to determine the current sample’s demographic distribution, respondents were asked to report their age, schooling, religiosity, socio-economic and relationship status.

### 3.4 Data analysis

**Figure 1:**
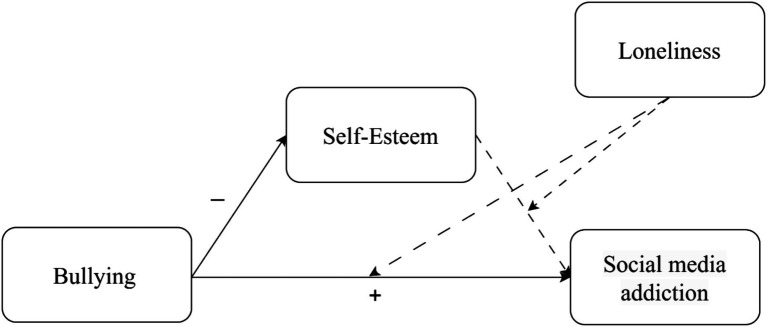
Gen X: a moderated mediation model. Continuous lines represent significant effects, while dashed lines represent non-significant effects.

**Figure 2:**
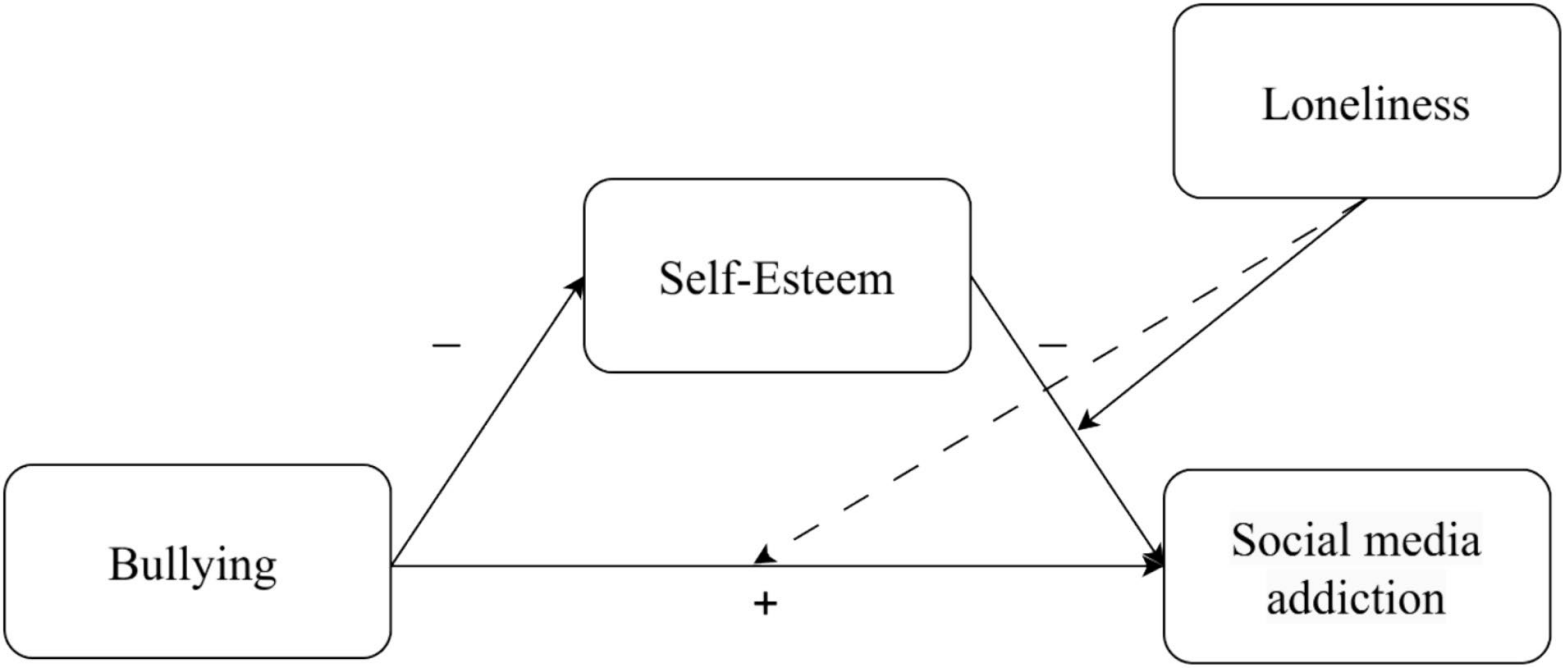
Gen Y: a moderated mediation model. Continuous lines represent significant effects, while dashed lines represent non-significant effects.

**Figure 3:**
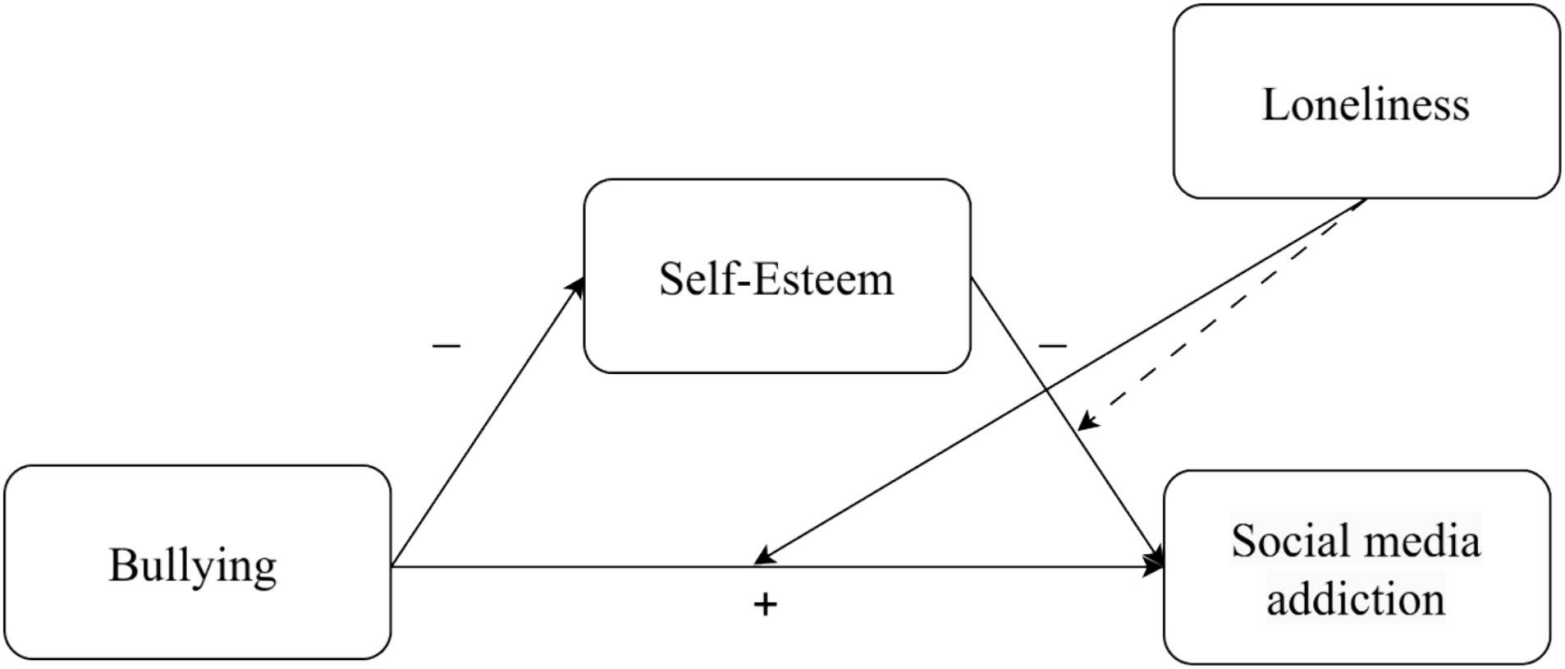
Gen Z: a moderated mediation model. Continuous lines represent significant effects, while dashed lines represent non-significant effects.

To test the research hypotheses the current study implemented a moderated mediation model-15 of the PROCESS v4.2 macro for SPSS (Hayes, 2017). Subjection to bullying as children or adolescents was positioned as the independent variable, self-esteem as the mediator, social media addiction as the dependent variable, and loneliness as the moderator in the relationship between self-esteem and social media addiction, and between subjection to bullying as children or adolescents and social media addiction (see Figures 1–3). The study utilized 10,000 bootstrap samples, establishing a 95% confidence interval. Additionally, moderation exploration involved estimating the conditional direct effects at three specific points: one standard deviation (SD) above the mean, at the mean, and one SD below the mean. Descriptive statistics, including means and standard deviations, were computed for all variables in this study, as outlined in Table 2.

**Table 2:** Means, standard deviations, and Pearson correlations of the research variables.

		1	2	3	*M* (SD)
Gen. X	1. Subjection to bullying in juvenile ages	–			31.56 (13.40)
	2. Self-esteem	−0.217^**^			23.30 (5.85)
	3 Loneliness	0.266^**^	−0.314^***^		3.91 (1.33)
	4. Social media addiction	0.257^**^	−0.213^*^	0.225^**^	10.30 (4.65)
Gen. Y	1. Subjection to bullying in juvenile ages	–			38.56 (18.76)
	2. Self-esteem	−0.392^***^			21.90 (5.44)
	3. Loneliness	0.274^***^	−0.476^***^		4.29 (1.46)
	4. Social media addiction	0.264^***^	0.301^***^	0.196^**^	12.59 (5.32)
Gen. Z	1. Subjection to bullying in juvenile ages	–			37.80 (18.53)
	2. Self-esteem	−0.277^***^			21.49 (5.21)
	3. Loneliness	0.360^***^	−0.371^***^		4.48 (1.42)
	4. Social media addiction	0.181^***^	−0.213^***^	0.169^***^	14.16 (5.12)

^*^p < 0.05, ^**^p < 0.01, ^***^p < 0.001.

## 4 Results

Within the Gen X cohort, hypothesis H1 was confirmed. As hypothesized, a positive association emerged between juvenile subjection to bullying and social media addiction. However, H2 was rejected; while a negative association surfaced between subjection to bullying as children or adolescents and self-esteem, no association was found between self-esteem and social media addiction. Therefore, self-esteem did not operate as mediator in the association between these variables. Moreover, loneliness, as hypothesized, did not serve as a moderator in the association between social media addiction and either bullying (H3a) or self-esteem (H3b) (see Figure 1).

Conversely, within the Gen Y cohort hypothesis H1 was confirmed. There was a positive association between subjection to bullying as children or adolescents and social media addiction. Moreover, in line with H2, it was established that self-esteem served as a mediator between these factors. Specifically, more intensive subjection to bullying was found to be negatively correlated with self-esteem, which in turn was negatively correlated with social media addiction. Loneliness moderated the negative association between self-esteem and social media addiction. However, in contrast to H4a, this association was more pronounced at lower levels of loneliness (SD = −1) compared to moderate levels of loneliness (SD = 0), but was not statistically significant at higher levels of loneliness (SD = +1) (see Figure 1). Also, in contrast to H4b, loneliness did moderate the association between bullying and social media addiction.

In the Gen Z cohort, hypotheses H1 and H2 were also confirmed. Self-esteem served as mediator in the positive association between subjection to bullying in juveniles and social media addiction, such that more intensive subjection to bullying was negatively correlated with men’s self-esteem, which in turn was negatively correlated with social media addiction. Also, in line with H5a, loneliness did not moderate the association between self-esteem and social media addiction. Loneliness moderated the positive association between subjection to bullying as children or adolescents and social media addiction. However, in contrast to H5b, this association was more pronounced at lower levels of loneliness (SD = −1) compared to moderate levels (SD = 0), but was not statistically significant at higher levels of loneliness (SD = +1) (see Figure 3).

For the regression coefficients and standard errors in the moderated mediation model, see Table 3. For conditional direct effects of self-esteem and subjection to bullying as children or adolescents on social media addiction at varying values of loneliness, see Table 4.

**Table 3:** Regression coefficients and standard errors in the moderated mediation model.

Gen X					
Antecedent variables	Consequent variables				
	*B*	SE	*t*	95%CI	*R* ^2^
Self-esteem (M)					
Subjection to bullying in juvenile ages	−0.095	0.04	−2.64^**^	[−0.166, −0.024]	0.05^**^
Social media addiction (Y)					
Subjection to bullying in juvenile ages	0.070	0.03	2.35^*^	[0.011, 0.128]	0.13^**^
Self-esteem	−0.114	0.07	−1.67	[−0.248, 0.021]	
Loneliness	0.230	0.33	0.69	[−0.428, 0.888]	
Subjection to bullying in juvenile ages × loneliness	−0.003	0.02	−0.16	[−0.039, 0.034]	
Self-esteem × loneliness	−0.080	0.04	−1.79	[−0.169, 0.008]	

Gen Y					
Antecedent variables	Consequent variables				
	*B*	SE	*t*	95%CI	*R* ^2^
Self-esteem (M)					
Subjection to bullying in juvenile ages	−0.112	0.02	−6.86^***^	[−0.145, 1.19]	0.15^***^
Social media addiction (Y)					
Subjection to bullying in juvenile ages	0.046	0.02	2.52^*^	[0.01, 0.08]	0.14^***^
Self-esteem	−0.240	0.07	−3.48^**^	[−0.38, −0.10]	
Loneliness	0.276	0.24	1.13	[−0.21, 0.76]	
Subjection to bullying in juvenile ages × loneliness	−0.005	0.01	−0.39	[−0.03, 0.02]	
Self-esteem × loneliness	0.083	0.04	2.16^*^	[0.01, 0.16]	

Gen Z					
Antecedent variables	Consequent variables				
	*B*	SE	*t*	95%CI	*R* ^2^
Self-esteem (M)					
Subjection to bullying in juvenile ages	−0.077	0.014	−5.53^***^	[−0.105, −0.050]	0.08^***^
Social media addiction (Y)					
Subjection to bullying in juvenile ages	0.040	0.02	2.56^*^	[0.01, 0.07]	0.08^***^
Self-esteem	−0.155	0.06	−2.80^**^	[−0.26, −0.05]	
Loneliness	0.336	0.21	1.60	[−0.07, 0.75]	
Subjection to bullying in juvenile ages × loneliness	−0.024	0.01	−2.61^**^	[−0.04, −0.01]	
Self-esteem × loneliness	−0.019	0.03	−0.56	[−0.08, 0.05]	

^*^p < 0.05, ^**^p < 0.01, ^***^p < 0.001.

**Table 4:** Conditional direct effects of self-esteem and subjection to bullying in juvenile ages on the social media addiction at varying values of loneliness.

Effects	*B*	SE	*t*	95%CI
Conditional direct effect of the self-esteem on social media addiction at values of loneliness (Gen X)				
Loneliness at −1 SD	N/A	N/A	N/A	N/A
Loneliness at 0 SD				
Loneliness at 1 SD				
Conditional direct effect of the subjection to bullying in juvenile ages on social media addiction at values of loneliness (Gen X)				
Loneliness at −1 SD	N/A	N/A	N/A	N/A
Loneliness at 0 SD				
Loneliness at 1 SD				
Conditional direct effect of the self-esteem on social media addiction at values of loneliness (Gen Y)				
Loneliness at −1 SD	−0.362	0.10	−3.71^***^	[−0.553, −0.170]
Loneliness at 0 SD	−0.240	0.07	−3.48^***^	[−0.376, −0.104]
Loneliness at 1 SD	−0.118	0.08	−1.48	[−0.275, 0.039]
Conditional direct effect of the subjection to bullying in juvenile ages on social media addiction at values of loneliness (Gen Y)				
Loneliness at −1 SD	N/A	N/A	N/A	N/A
Loneliness at 0 SD				
Loneliness at 1 SD				
Conditional direct effect of the self-esteem on social media addiction at values of loneliness (Gen Z)				
Loneliness at −1 SD	N/A	N/A	N/A	N/A
Loneliness at 0 SD				
Loneliness at 1 SD				
Conditional direct effect of the subjection to bullying juvenile ages on social media addiction at values of loneliness (Gen Z)				
Loneliness at −1 SD	0.077	0.02	3.26^**^	[0.030, 0.123]
Loneliness at 0 SD	0.052	0.02	2.99^**^	[0.018, 0.087]
Loneliness at 1 SD	0.003	0.02	0.17	[−0.033, 0.039]

^*^p < 0.05, ^**^p < 0.01, ^***^p < 0.001.

## 5 Discussion

The current study aimed to explore the interconnectedness of juvenile bullying, self-esteem, loneliness, and social media addiction among men across three generational cohorts.

### 5.1 Discussion of general findings

In line with Social Learning theory, we found significant positive relationships between juvenile bullying and social media addiction in adulthood. The positive association between bullying and social media addiction in all generational cohorts suggests that the coping mechanisms and behaviors learned from different bullying experiences may persist over time, leading to compulsive adult behavior in digital environments. This expands the theory by applying it to digital and adult contexts, showing that learned behaviors from childhood experiences continue to influence adult personal and social life through the life span in very different online settings and beyond cultural and historical backgrounds of different generational cohorts.

In line with Self-Concept theory (Rogers, 1959), we found a negative association between bullying victimization in juveniles and self-esteem in adulthood in all three generations. This finding underscores the profound and enduring impact that past bullying experiences have on individuals, persisting into adulthood across multiple generations. It highlights the universal nature of bullying’s traumatic effects, demonstrating how such early adversities can deeply shape and lower self-esteem, irrespective of societal and technological changes over time (Schäfer et al., 2004).

### 5.2 Discussion of findings in three generational cohorts

However, the other findings of our study emphasize differences between generational cohorts, which can be explained through three major shifts, reflecting social and cultural developments, and defining the three cohorts. The first shift reflects the *evolution of masculinity*: from traditional hegemonic masculinity (Connell et al., 1985) in Gen X to more flexible non-traditional masculinity in Gen Y (Green and McClelland, 2019) and hybrid masculinity (Huang et al., 2023) in Gen Z, which extensively broadens the scope of masculine behaviors and emotional expressions. The second shift is reflected in the *type of bullying* experienced by the participants in their childhood: from only traditional forms of bullying (physical and verbal) in Gen X, mostly traditional with the emergence of cyberbullying in Gen Y to both traditional and cyberbullying in Gen Z (Hinduja and Patchin, 2014). The third shift is much more compressed in time and reflects both the *evolution of social media* and *generational needs and gratifications* (Laor and Galily, 2022; Lissitsa, 2024; Zimand-Sheiner and Lissitsa, 2024).

*Generation X.* The findings among Gen X show that subjection to bullying in juveniles was positively associated with social media addiction, but negatively associated with self-esteem. However, self-esteem did not serve as a mediator in the association between these variables. Additionally, loneliness did not moderate the associations between social media addiction and either subjection to bullying or self-esteem. The possible explanation may lie in the fact that Gen X are digital immigrants (Çoklar and Tatli, 2021) who adapted to social media and digital communication as adults rather than growing up with it. Their relationship with social media is likely more functional and less integrated into their identity compared to later generations. Their self-concept was formed prior to the ubiquity of digital life, making their self-esteem less intertwined with their online presence. During Gen X’s youth, bullying predominantly took traditional forms—physical and verbal—without the pervasive nature of cyberbullying (Hinduja and Patchin, 2014). This meant that while bullying had an immediate and significant impact, it was restricted to specific environments and times (e.g., during school hours or in specific social settings). The limited scope and exposure to bullying might have led to a more compartmentalized impact on their lives, potentially influencing their self-esteem negatively but not to the extent that it became a pervasive factor in their adult lives. The alternative explanation may be that the impact of bullying on Gen X could still have had profound consequences, but not necessarily leading to digital escapism due to their later introduction to digital platforms. It’s plausible that for men of this generation, the repercussions of diminished self-esteem from such experiences might manifest themselves through more conventional means such as withdrawal, alcohol use, violence, and various addictions, rather than an inclination toward social media for relief (Bourdon et al., 2020). Future studies addressing different types of addictions might clarify this point. The adherence to the traditional masculine norms of Gen X (Wagner and Reifegerste, 2024) might explain why loneliness did not emerge as a significant moderator. Despite any feelings of loneliness, Gen X men might have been less inclined to acknowledge or address such feelings, especially in a public forum like social media, due to the prevailing masculine ideals.

*Generation Y.* The findings among Gen Y show the mediation effect of self-esteem in the association between subjection to bullying in juveniles and social media addiction, and the moderation effect of loneliness in the relationships between self-esteem and social media addiction. Gen Y experienced a mix of traditional bullying and the early stages of cyberbullying (Hinduja and Patchin, 2014). This exposure to both physical and emerging digital forms of bullying might have led to more complex and enduring impacts on their psychological development, including their self-esteem. Their bullying experiences were not only physically present, but could also extend into their online lives, creating a more continuous and pervasive impact (Kwan et al., 2020). In terms of Self-Concept Theory (Rogers, 1959), bullying victimization caused incongruence between their perceived self and ideal self, the latter of which can be created in social media profiles, and encouraged compulsive social media use (Kaplan et al., 2023). Gen Y men who suffered from bullying as juveniles may turn to platforms like Facebook and Instagram, focusing on personal life, achievements, and social connectivity, to seek the validation and social connections they felt deprived of in their real lives.

We found that in Gen Y, loneliness moderated the relationship between self-esteem and social media addiction, but this was more pronounced at lower levels of loneliness. This suggests that for Gen Y, even those with relatively fulfilling social lives (indicated by lower levels of loneliness) might still turn to social media as a means of coping with the residual effects of bullying on their self-esteem. The shift in masculinity norms from traditional hegemonic masculinity toward more flexible and emotionally expressive masculine ideals could explain why even those with lower levels of loneliness (who are presumably more open to emotional expression and connection) might still heavily rely on social media to manage self-esteem issues stemming from past bullying. The lack of moderation by loneliness in the relationship between bullying and social media addiction could indicate that the use of social media as a coping mechanism for past bullying is a consistent behavior in Gen Y, regardless of their current level of loneliness.

*Generation Z.* The findings among Gen Z show the mediation effect of self-esteem in the association between subjection to bullying as juveniles and social media addiction, and the moderation effect of loneliness in the relationships between bullying and social media addiction, while the moderation effect of loneliness in the relationship between self-esteem and social media addiction was not found to be significant. Gen Z has been exposed to both traditional forms of bullying and cyberbullying (Hinduja and Patchin, 2014). The pervasive nature of cyberbullying, facilitated by constant digital connectivity, means that Gen Z might experience bullying as a more continuous and inescapable part of their lives, impacting their self-esteem more profoundly. Dealing with incongruence between their perceived self and ideal self encourages them to process their experiences, reclaim their narratives, and present themselves in a manner that contrasts with the disempowerment they felt during bullying incidents. The complex dynamics of hybrid masculinity could contribute to Gen Z men’s approach to dealing with bullying-related self-esteem issues, possibly making them more open to seeking validation and support through social media platforms. Engaging in creative expression and content creation on digital platforms such as TikTok and YouTube offers a therapeutic outlet. By transitioning from the role of a bullying victim to that of a content creator, Gen Z individuals exert agency over their digital and social identities. The positive feedback loop created by likes, comments, and shares serves as a form of social inclusion and validation that directly counteracts the negative self-perceptions stemming from bullying. This increased engagement, while initially beneficial for self-esteem, can become compulsive over time, leading to social media addiction (Vaillancourt-Morel et al., 2020). Even those with lower levels of loneliness—who might be expected to have sufficient offline social support—find value in the unique forms of expression and community these platforms offer. They may still turn to these platforms to reclaim agency over their narrative and to engage with a community that resonates with their experiences and interests, to explore and express their identities in ways that align with contemporary masculinity. The lack of moderation by loneliness in the relationship between self-esteem and social media addiction could suggest that for Gen Z, the use of social media as a coping strategy for self-esteem issues is a widespread behavior, independent of their current loneliness levels.

## 6 Conclusion

In sum, our study makes an important contribution to the Generational Cohorts Theory by elucidating how the intersection of bullying, self-esteem, loneliness, and social media addiction is manifested distinctly across Gen X, Gen Y, and Gen Z. This differentiation is intricately tied to the evolution of masculinity, the nature of bullying encountered during formative years, and the generational engagement with social media platforms. This approach not only enhances theoretical understanding but also highlights the unique challenges and coping methods of each group, especially regarding bullying and its harmful impact on self-esteem. The aim goes beyond meeting generational needs; it’s about creating strong, precisely aimed prevention, intervention, and treatment methods capable of identifying, diagnosing, and managing these issues effectively. Ultimately, this study emphasizes the importance of implementing strategies that actively confront and reduce the significant challenges of bullying and low self-esteem across generations.

## 7 Study limitations and directions for further research

The current study has several limitations. We used convenience sampling, which may impact the generalizability of our findings. A notable limitation of our study stems from its cross-sectional design, wherein the assessment of the bullying experiences relied on respondents’ recollections of their past rather than capturing these experiences in real time. This approach introduces the potential for recall bias, as participants’ memories may be influenced by subsequent events or their current psychological state, potentially affecting the accuracy and reliability of the reported bullying incidents. Future research would benefit significantly from adopting a longitudinal design. Such an approach would enable the tracking of juvenile bullying experiences and their psychological impacts over time, providing a more accurate and dynamic understanding of how these experiences influence self-esteem, loneliness, and social media addiction from juvenile age into adulthood. This would not only mitigate the limitations associated with reliance on retrospective accounts, but also offer deeper insights into the causal relationships and evolving nature of these variables across different life stages. Additionally, our focus was exclusively on men without conducting a comparative analysis with women. Therefore, whether the observed patterns and models are truly distinct between genders remains unclear. Future research should aim to include both men and women in a randomized sampling method to explore these dynamics further and assess the specificity of our findings across different genders.

## Data availability statement

The raw data supporting the conclusions of this article will be made available by the authors, without undue reservation.

## Ethics statement

The studies involving humans were approved by Institutional Ethics Committee for Non-Clinical Human Studies at the authors’ university (AU-SOC-MK-20221031). The studies were conducted in accordance with the local legislation and institutional requirements. The participants provided their written informed consent to participate in this study. Written informed consent was obtained from the individual(s) for the publication of any potentially identifiable images or data included in this article.

## Author contributions

SL: Writing – original draft, Conceptualization. MK: Writing – original draft, Supervision, Project administration, Methodology, Investigation, Formal analysis, Data curation, Conceptualization.
